# Potential for non-combustible nicotine products to reduce socioeconomic inequalities in smoking: a systematic review and synthesis of best available evidence

**DOI:** 10.1186/s12889-019-7836-4

**Published:** 2019-11-06

**Authors:** Mark Lucherini, Sarah Hill, Katherine Smith

**Affiliations:** 1 Global Health Policy Unit, School of Social & Political Science, University of Edinburgh, Edinburgh, UK; 2 School of Geography, Geology and the Environment, Keele University, Newcastle, UK; 3 School of Social Work & Social Policy, University of Strathclyde, Glasgow, UK

**Keywords:** E-cigarettes, Smokeless tobacco, Nicotine replacement therapy, Inequalities

## Abstract

**Background:**

While some experts have emphasised the potential for e-cigarettes to facilitate cessation among smokers with low socioeconomic status (SES), there is limited evidence of their likely equity impact. We assessed the potential for electronic cigarettes and other non-combustible nicotine-containing products (NCNPs) to reduce inequalities in smoking by systematically reviewing evidence on their use by SES in countries at stage IV of the cigarette epidemic.

**Methods:**

Ten electronic databases were searched in February 2017 using terms relating to e-cigarettes, smokeless tobacco and nicotine replacement therapy (NRT); and SES. We included studies published since 1980 that were available in English and examined product use by SES indicators such as income and education. Data synthesis was based on those studies judged to be of medium- to high-quality using guidelines adapted from the Critical Appraisal Skills Programme.

**Results:**

We identified 54 studies describing NCNP use by SES across 12 countries, of which 27 were judged of sufficient quality to include in data synthesis. We found mixed patterns of e-cigarette current use by SES, with evidence of higher use among low-income adults but unclear or mixed findings by education and occupation. In contrast, smokeless tobacco current use was consistently higher among low SES adults. There was very limited evidence on the SES distribution of NRT in adults and of all NCNPs in young people.

**Conclusions:**

The only NCNP for which there are clear patterns of use by SES is smokeless tobacco, where prevalence is higher among low SES groups. While this suggests a potentially positive impact on inequalities in smoking (if NCNP use displaces smoked tobacco use), this has not been seen in practice. These findings do not support the suggestion that e-cigarettes have the potential to reduce social inequalities in smoking, since i) current evidence does not show a clear trend of higher e-cigarette use in population groups with higher tobacco consumption, and ii) the experience of smokeless tobacco suggests that – even where NCNP use is higher among low SES groups – this does not necessarily replace smoked tobacco use in these groups.

## Introduction

While the prevalence of combustible tobacco smoking has decreased over recent decades in many high-income countries [1], cigarette use continues to be higher among groups experiencing social disadvantage [2]. Such inequalities have been observed across numerous measures of social location – including ethnicity, gender and sexual orientation – with the most extensive evidence found in relation to socioeconomic status (SES) [3]. There is therefore an urgent need to understand what may help reduce these inequalities.

Recent reviews focussing on population (e.g. taxation, smoke-free environments) and individual-level tobacco control interventions have found limited evidence of measures likely to help reduce the SES gradient in smoking, with the exception of raising tobacco prices (via taxation) [4, 5]. The growing use of e-cigarettes has led to speculation that these may offer an alternative to smoked tobacco among those who struggle to quit, including smokers from lower socioeconomic groups [6, 7] . This expectation is reflected in calls to improve their accessibility for disadvantaged smokers [8].

While a number of systematic reviews have sought to assess e-cigarettes’ potential as a cessation aid, the relatively nascent evidence base makes it difficult to draw firm conclusions [9–14]; and there is even less evidence to indicate how e-cigarettes may affect smoking inequalities [8]. A previous review by Hartwell et al. [15], which sought to assess whether e-cigarette awareness, ever use and current use varied between different sociodemographic groups, found greater e-cigarette awareness and ever-use among those with higher education, but no clear patterns in terms of other SES indicators (income and occupation) or for current e-cigarette use. The authors suggest that – consistent with diffusion of innovation theory [16] – early adopters tend to come from more privileged social groups, and raise the possibility that e-cigarettes might initially be expected to widen smoking inequalities, assuming they encourage smoking cessation. The diffusion of innovation theory is supported by data from the UK indicating e-cigarette use has become more equally spread by SES over time [17], although this pattern is not evident in the US population [18]. Our review extends Hartwell et al’s [15] through the inclusion of more recent evidence (published since 2015) and through comparing the emerging trends of e-cigarettes use with those of more established non-combustible nicotine-containing products (NCNPs): smokeless tobacco and nicotine replacement therapy (NRT).

While e-cigarettes offer a novel form of nicotine delivery, other NCNPs have previously attracted interest from a harm reduction perspective and experience with these products may offer an indication of the future potential of e-cigarettes. Pharmaceutical NRT is the only NCNP to have received widespread acceptance within the tobacco control community, although primarily as an aid to cessation rather than a long-term nicotine substitute [19]. Meanwhile, smokeless tobacco is a non-pharmaceutical NCNP that has been positioned by some as a potential aid to quitting or reducing cigarette use [20].

Smokeless tobacco use is often culturally driven compared to the mostly harm reduction/cessation driven use of NRT. In Norway and Sweden, snus has long been part of a normalised cultural practice [21]. Smokeless tobacco is also available in the US and Canada, where it is often prevalent among men in rural communities [22] and its uptake is sometimes perceived as a rite of passage for young men into maturity [23, 24]. Despite this cultural perception of smokeless tobacco, it is still often positioned as a smoking alternative. Lund credits smokeless tobacco use for reducing rates of smoking among men in Norway [21] and Levy et al. note that smokeless tobacco is likely 90% less harmful than combustible tobacco [25] (not dissimilar to Public Health England’s recent estimate for e-cigarettes [26]). There are therefore, emerging parallels between e-cigarettes and smokeless tobacco use. Qualitative research also reveals user perceptions of e-cigarettes to be split between recreational and medical uses [27, 28]. Other research indicates an emerging vaping ‘sub-culture’ [29], revolving around specialised expertise and ‘tricks’ [30]. Like smokeless tobacco, e-cigarettes have also been correlated with smoking cessation but causation is yet to be determined [31].

While there are cultural and cessation similarities between e-cigarettes and smokeless tobacco, there are also legislative similarities in the sense that legislation for both products can be contentious, and, especially for e-cigarettes, quite fluid. In the US, for instance, public health bodies are pushing for increased regulation of e-cigarettes [32, 33], while smokeless tobacco remains widely available [34]. Conversely in the UK, public health bodies are increasingly pushing towards relaxed regulation for e-cigarettes [35, 36], but, like most of Europe, smokeless tobacco is highly regulated [37]. Norway and Sweden remain the only European countries where smokeless tobacco is widely available [21].

It is therefore, potentially useful to compare emerging patterns of e-cigarette use by SES with those of smokeless tobacco. The case for comparing e-cigarettes with NRT is perhaps less clear, given NRT’s firm position as a medical product. Nicotine-replacement therapy was only briefly subject to minimal regulation before being classed as a medically-licenced product and enlisted into harm reduction strategies in most jurisdictions [38]. However, NRT use is not always straightforwardly associated with quitting smoking with one study finding that one-third of users across the US, UK, Canada and Australia used NRT for a reason other than quitting, including cutting down or temporary abstinence [39]. Qualitative studies further suggest social reasons underlying NRT use including being able to use it (or not) discreetly, advice from health professionals, and general inconsistency of knowledge about the product [40]. While the positioning of the three products is distinct they also share many similarities, one of the foremost being that research into patterns and norms of use remains underexplored.

The aim of this review is to assess the potential for NCNPs to reduce inequalities in smoking by systematically reviewing evidence on their use by SES in countries at stage IV of the cigarette epidemic. Given the complex NCNP landscape discussed above, the review aims to evaluate the population-level equity impacts of NCNP, rather than focussing on their use as cessation aids. Therefore, the review will not try to distinguish between those using NCNP for recreation or for cessation/harm reduction. The synthesis of included studies will assess potential equity impacts of e-cigarettes alongside other NCNPs, independent of other interventions (such as behavioural cessation support). This will be done by seeking evidence on each product’s distribution and (where available) impact by SES in countries at stage IV of the cigarette epidemic (i.e. those with strong tobacco control policies, declining overall prevalence in both men and women, and higher smoking prevalence in lower socioeconomic groups [1]). These countries are most relevant to the research aim on the basis of this distribution of tobacco use by SES [2, 3] and therefore positive, negative or other equity impacts are more easily discernible. Countries will not be defined by their approach to cessation support or regulation of NCNP, which is outside the scope of this review. We defined NCNPs as products delivering nicotine in the absence of combustible tobacco; and consider SES as a person’s position in the social and economic structure of society, represented by indicators such as education, occupation and household income [41].

## Methods

The review is reported in line with Preferred Reporting Items for Systematic Reviews and Meta-Analyses (PRISMA) Equity guidelines (Additional file 1), and a full protocol is registered with PROSPERO (ID: CRD42017080672) [42].

### Search strategy

A search string combining terms for NCNP, socioeconomic inequalities and combustible tobacco smoking (Additional file 2) was used to search ten databases on 9th February 2017: BIOSIS Citation Index, Web of Science, Cochrane Library, ProQuest Social Sciences, CINAHL Plus, EMBASE, Medline (+ Medline Epub ahead of print), PsycInfo, Global Health. An initial 24,711 studies with English language abstracts were identified across all databases.

### Study selection and eligibility criteria

We limited the review to publications from 1980 onwards, reflecting the emergence of both socioeconomic inequalities in smoking [43] and harm reduction approaches [44] from around that date. No limits were set on study design but we restricted our database search to articles available in English. We included all studies that might provide evidence on the differential impact of NCNP by SES for full-text review. We also identified nine qualitative studies examining NCNP in low SES groups which are considered in a separate report.

To meet out inclusion criteria, studies had to report the prevalence of NCNP use by an appropriate SES indicator in a representative population or sample. In order to improve generalisability for examining equity impacts by SES, we excluded studies that focused on specific population sub-groups (patient populations, ethnic sub-groups, specific occupational groups), although we included studies limited by sex or age group. We also excluded studies where it was not possible to distinguish use of NCNP in isolation from other interventions (such as behavioural cessation support), and those from countries not at stage IV of the cigarette epidemic [1].

**Fig. 1:**
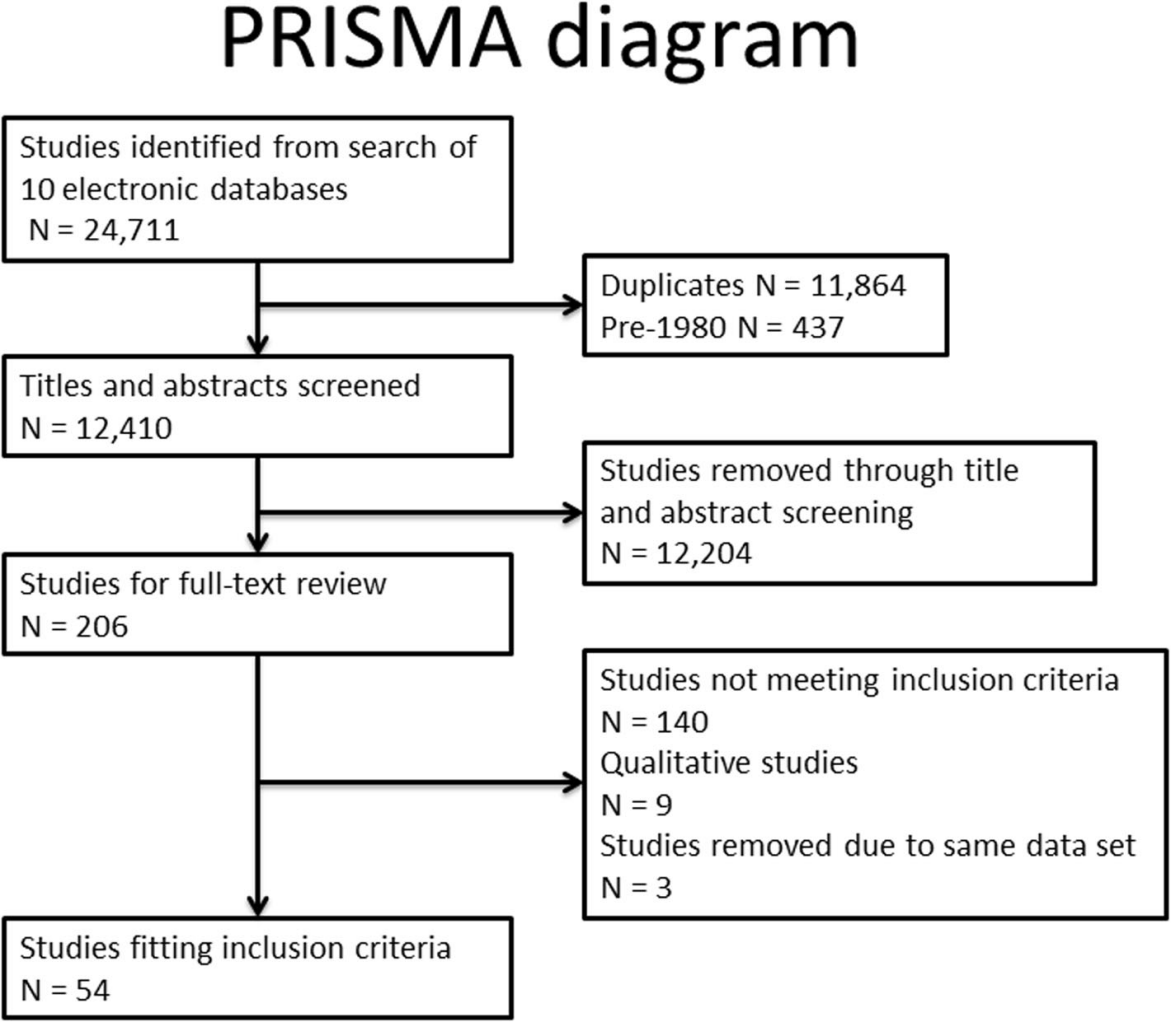
PRISMA flow diagram

Following initial screening and removal of obviously irrelevant references, ML assessed titles and abstracts against eligibility criteria. Full text review was carried out for 206 studies, including full independent assessment of 25% by two authors and secondary review of all exclusions. Discrepancies were resolved by discussion between two or more authors. Where more than one article was published on the basis of the same study data, we selected the article that scored higher on our quality assessment (described below). Our final evidence base comprised 54 unique studies (Fig. 1).

### Data extraction and quality assessment

We developed a data extraction and quality appraisal template to capture relevant study details and facilitate quality assessment (Additional file 3); this was piloted with a small number of studies (c.5) before being finalised and applied to all studies. Quality appraisal was based on relevant Critical Appraisal Skills Programme tools [45] adapted to the focus of this review. This appraisal was based on various criteria including approaches to data analysis and consideration of confounding factors (see Additional file 5 for full details). Our assessments suggested a high proportion of low quality evidence, with the main limitations being a lack of power to detect differences by SES, imprecise measures of NCNP use, and lack of age-adjustment; these limitations often made it impossible to interpret the study findings with respect to our review question. We therefore decided to undertake a ‘best evidence’ review [46] in order to focus on the most relevant evidence that met a minimum quality threshold. Overall quality was assessed as high, medium or low. Higher ratings were given to studies reporting the prevalence of NCNP use by SES with adequate power to detect differences by SES.

### Data synthesis

The review findings were summarised via narrative synthesis according to type of NCNP. For adults, synthesis of findings was based on studies of medium or higher quality that reported the distribution of NCNP use by SES (Additional file 4). We synthesised findings separately for young people as patterns of tobacco use by traditional SES indicators are less consistent among this age group, who also tend to transition in and out of tobacco use [47, 48]. For young people, we therefore included only studies that provided evidence on both NCNP and cigarette use by SES in the same population (Additional file 4). Meta-analyses were not possible or appropriate given the diversity of study measures and the heterogeneity of study settings.

**Fig. 2:**
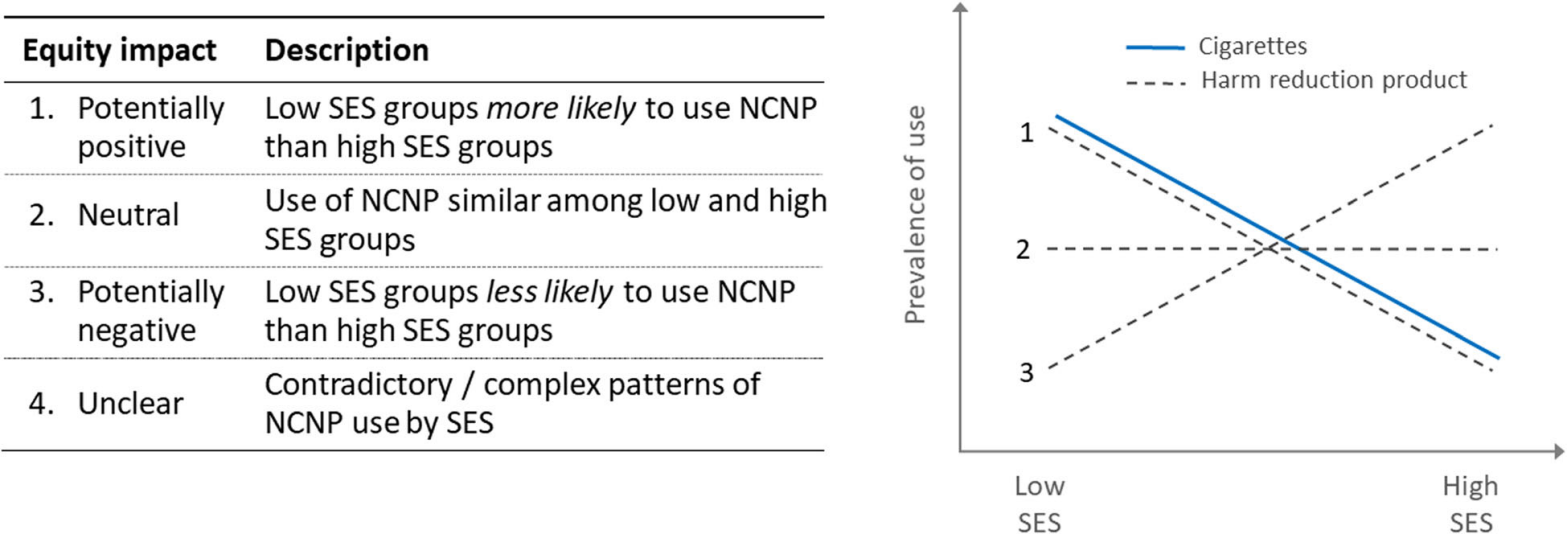
Predicted equity impact

In order to clearly summarise a complex evidence base while addressing our research aim, we extrapolated possible equity outcomes from each of our ‘best evidence’ studies as suggested by the study findings (Fig. 2). Potentially positive impacts were identified when use of NCNP was higher among low SES compared with high SES groups (line 1 in Fig. 2). Conversely, potentially negative impacts were identified when use of NCNP was lower among low SES groups (line 3). When use of NCNP was roughly equal by SES (line 2), this was regarded as representing an equity-neutral impact. Finally, contradictory or complex patterns of NCNP use were classed as unclear with respect to expected equity impact.

It should be noted that these classifications represent the *potential* equity impact of NCNPs, based on three assumptions. First, they assume that NCNP use will lead to a decline in combustible tobacco consumption (and thus a harm reduction effect). While this is a reasonable assumption in relation to NRT (which is used primarily as an aid to smoking cessation), its applicability to e-cigarettes and smokeless tobacco is less clearly established. Second, we assumed that conventional cigarette smoking in the relevant population is more prevalent in low compared with high SES groups (solid line in Fig. 2). This is a relatively safe assumption for adults, given well-established patterns of smoking in Stage IV countries [1–3]; however the assumption may not hold for young people, reflected in our decision to limit ‘best evidence’ for this age group to studies reporting the distribution of smoking as well as NCNP use by SES. Third, we assumed that the distribution of NCNP use by SES in the general population would mirror that among smokers. This assumption is open to criticism, since i) not all NCNP users will be smokers or ex-smokers; and ii) smokers will have a less advantaged socioeconomic profile compared with the general population. Nevertheless, with these assumptions in mind, we felt this approach was the most appropriate way of trying to clearly summarise a complex evidence base.

Our synthesis summarises the distribution of best available evidence for the potential equity impact of each NCNP (e-cigarettes, smokeless tobacco and NRT) for adults and young people. Summary figures (compiled for e-cigarette and smokeless tobacco use in adults) depict NCP current and ever use by each available measure of SES (e.g. income, education, occupational group), such that studies that assessed use by more than one SES indicator contributed multiple measures to the evidence synthesis.

## Results

We identified 54 unique studies describing NCNP use by SES across 12 countries at stage IV of the smoking epidemic (see Additional file 7), with a majority of studies (35) located in the USA. The distribution of studies by product and country can be seen in Table 1. Nineteen studies focused exclusively on e-cigarettes, and 28 exclusively on smokeless tobacco (including snus). Three studies reported on e-cigarettes and smokeless tobacco. We identified only four studies focused on NRT (Additional file 6), and no eligible studies on other types of NCNP. All studies reported NCNP use by SES based on cross-sectional data, most often from surveys but in a few cases using baseline data from longitudinal studies. Indicators of SES included measures based on income, education, occupational group, composite indices and neighbourhood disadvantage. Measures of NCNP use included current use and ever use, with both sometimes used in the same study. Thirteen studies (five for e-cigarettes and eight for smokeless tobacco) had study populations ranging from 10 to 20 years old and were analysed separately from adult studies (those with populations 16 years and over). One study with an age range of 14–31 years and mean age of 19.5 was considered with the young people studies [49]. Definitions of ‘current’ and ‘ever use’ were not always consistent, but ‘current use’ most often referred to daily use, regular use or any use within the last 30 days; while ‘ever use’ was defined variously from any use in the past 12 months to ever lifetime use.

**Table 1:** Distribution of all 54 included studies, by NCNP type, country and age grouping. Three studies reported more than one NCNP, and so are counted twice in this table, hence the overall total of 57. Blank cells indicate no studies

	E-cigarettes		Smokeless tobacco		NRT		Total
	Best Evidence	Other	Best Evidence	Other	Best Evidence	Other	
US	5	8 + 1(YP)	13	7 + 3(YP)		1	38
Australia					1		1
Canada		1(YP)				1	2
Ireland		1(YP)					1
Italy		1					1
Finland		1(YP)					1
France		1(YP)					1
Norway			1 + 2(YP)	2(YP)			5
Sweden			2				2
Switzerland		1		1(YP)			2
UK	2				1		3
Total Adults	7	10	16	7	2	2	44
Total Young People	0	5	2	6	0	0	13
Total	7	15	18	13	2	2	57

### E-cigarettes

Seventeen studies examined e-cigarette use by SES in adults, of which seven met our definition of ‘best evidence’ (see Additional file 6). No study included details on the type of e-liquid used, i.e. whether or not this contained nicotine. For all adult e-cigarette studies that provided relevant data, combustible tobacco use was higher among low SES groups – thus confirming our first assumption. The data collection period for the best evidence studies was 2010–2014.

#### Current use among adults

**Fig. 3:**
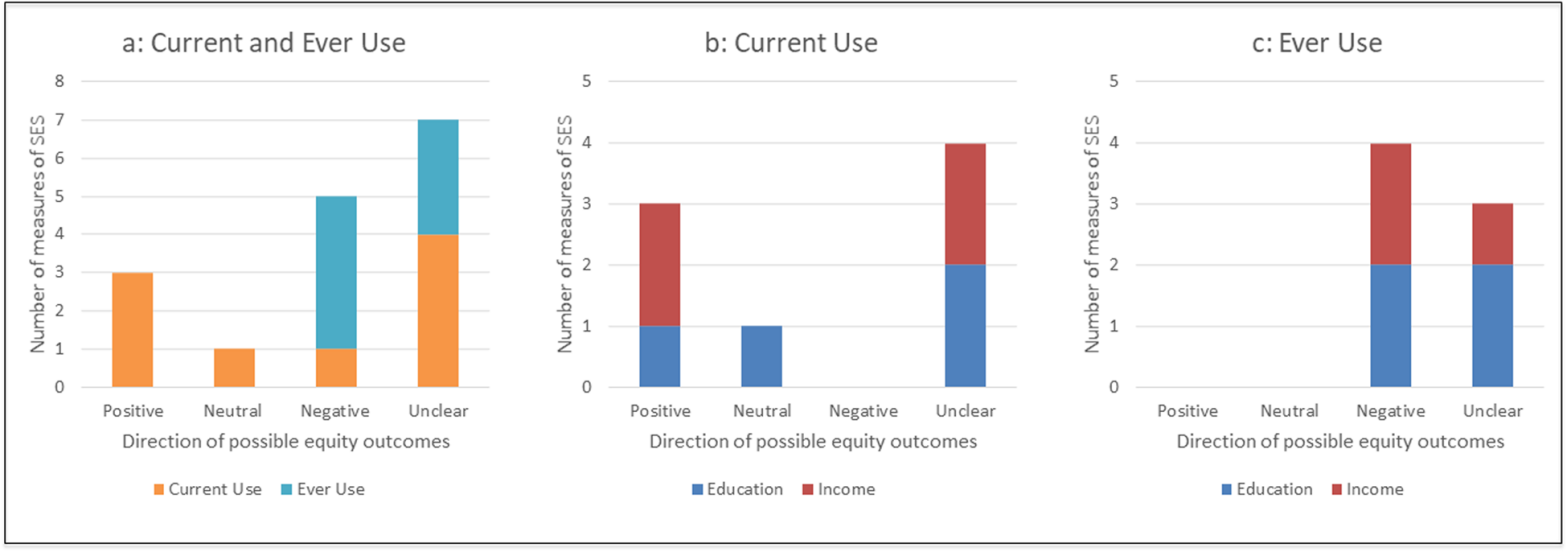
Potential equity impacts of e-cigarette use among adults. Current and ever use are presented together and then separately to provide a sense of difference in product use by the two different measures of education and income. **a** Current and ever use; **b** Current use only; **c** Ever use only

Evidence of the potential equity impacts of current e-cigarette use was somewhat inconsistent, but generally pointed towards positive equity impacts (Fig. 3, b). For instance, of four US studies using large population-based samples, two [50, 51] found potentially positive impacts for current use by income, but unclear or neutral impacts by education; while one [52] found the reverse (i.e. potentially positive impacts by education but unclear impacts by income). The fourth US study suggested unclear impacts [53]. A fifth study (from the UK) found potentially negative impacts of current e-cigarette use by occupational group [54]; while a sixth study (based on a small sample of e-cigarette users in the US) showed unclear impacts [55].

#### Ever use among adults

Studies reporting on e-cigarette ever use among adults showed a more consistent pattern, with potentially negative and unclear findings dominating (Fig. 3c). Two studies from the US [55, 56] indicated potentially negative equity impacts by income, with one showing unclear impacts by education; another US study indicated unclear outcomes by education and income [52]. Potentially negative impacts by both education and income were also found in a study among UK smokers [57].

#### Low quality studies, adults

Lower quality studies that were excluded from our ‘best evidence’ synthesis [58–67] showed broadly similar patterns to those illustrated above for both current and ever use of e-cigarettes, with many finding no clear pattern by SES.

#### Young people

Of the five studies examining e-cigarette use by SES in young people, none met our criteria for ‘best evidence’. Of three studies examining current use, two studies found potentially positive impacts (one from Finland examining use by education [68], and another from Ireland examining use by neighbourhood deprivation [69]) while a third study (from the US) found unclear impacts by education [70]. For e-cigarette ever use, the first two studies showed unclear findings [68, 69], as did a fourth study from France [71]. A fifth study (from Canada) found potentially positive impacts for ever use [72]. Overall, the limited quality and quantity of the evidence means no conclusions can be drawn on the likely equity impact of e-cigarette use among young people.

### Smokeless tobacco

Twenty-three studies examined smokeless tobacco use in adults by SES, of which 16 met our criteria for ‘best evidence’. For all but one of these studies, combustible tobacco use was higher among lower SES groups. Three studies reported on older data (1971–1990) [73–75], with the other 13 studies reporting data from the last 15 years (2005–2014). The majority (13) of studies came from the US, with two studies from Sweden and one from Norway. The ban on smokeless tobacco throughout the EU (except Sweden) explains the scarcity of European evidence on this form of NCNP.

#### Current use among adults

**Fig. 4:**
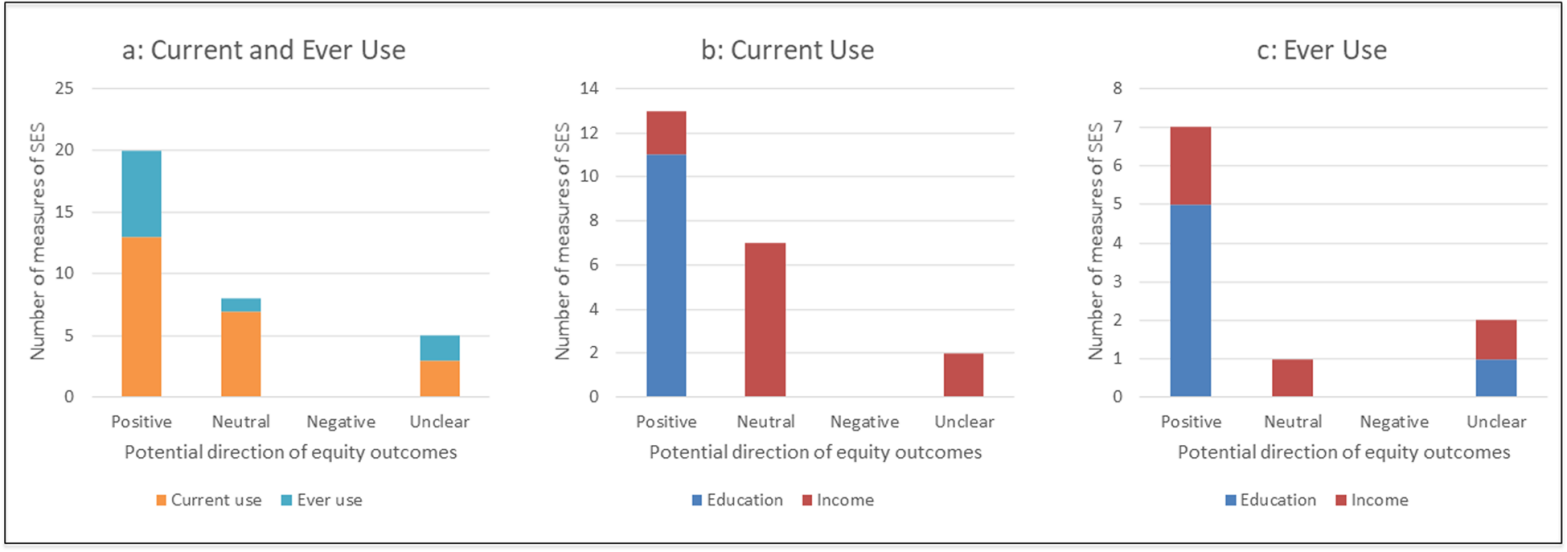
Potential equity impacts of smokeless tobacco use among adults. Current and ever use are presented together and then separately to provide a sense of difference in product use by the two different measures of education and income. **a** Current and ever use; **b** Current use only; **c** Ever use only

Studies from the USA and Scandinavia showed broadly similar patterns in the distribution of smokeless tobacco use by SES, with higher use among low SES groups suggesting a potentially positive equity impact (Fig. 4b). Studies of smokeless tobacco prevalence among large population-based samples came exclusively from the US; these indicated potentially positive impacts for current use by education, but mostly neutral or unclear impacts by income [51, 74, 76–83]. A study from Sweden [84], provided data on the association between smokeless tobacco use and SES (measured by education and income), but no prevalence data. Similar to the US evidence, this study indicated potentially positive impacts of smokeless tobacco use by education but unclear impacts by income. All studies found very low smokeless tobacco use among women compared with men, such that these findings effectively represent only male use of smokeless tobacco. Indeed, three of the 16 studies included only men in their study populations [74, 78, 82].

#### Ever-use among adults

A majority of studies looking at ever use - including four from the US and one from Norway - similarly suggested mostly positive equity impacts for smokeless tobacco use by education, and a mix of potentially positive, neutral or unclear impacts by income [73, 74, 85–87] (Fig. 4c). Interestingly, the study from Norway [86] focussed solely on women and was able to generate reliable data due to the relatively high use of snus among this population in Norway. A sixth study examining ever-use of snus among older adults (40–60 years) in Sweden [75] found unclear equity impacts by education.

#### Low quality studies, adults

A further six US studies were not included in our ‘best evidence’ synthesis due to limited quality. Two of these also suggested positive impacts from smokeless tobacco use, [88, 89] with the remaining four showing neutral or unclear patterns in use by SES [22, 58, 66, 90, 91].

#### Young people

Of eight studies examining smokeless tobacco use in young people only two met our ‘best evidence’ criteria, both conducted with secondary school students in Norway. One suggested higher smokeless tobacco current use among students with lower educational trajectories, but no clear pattern in relation to family income [92]; while the other found no gradient in smokeless tobacco current use by education [93]. Of the remaining six studies (of low quality or relevance), most showed unclear [94–97] or potentially positive equity impacts for current smokeless tobacco use [49, 98], although one suggested potentially negative equity impacts [99].

### Nicotine replacement therapy

We found only four eligible studies of NRT use by SES (all on adults), of which just two met our criteria for best evidence. Both studies drew on surveys of adult smokers. One, a UK study based on national survey data, indicated a neutral equity impact by occupational group among smokers who had used NRT in quit attempts over the previous year [100]. The other study [101] used data from four waves of an Australian national survey, and reported NRT use for cessation in the previous year; this indicated potentially negative equity impacts by income, but unclear impacts by education. The two low quality studies included one from Canada [102] suggesting neutral equity impacts by education and income; and another from the US [103] suggesting potentially positive impacts by education.

## Discussion

Our review confirms a lack of direct evidence on the likely impact of e-cigarettes on inequalities in smoking, and relatively limited evidence on the equity impact of other NCNP. This deficit is perhaps understandable for e-cigarettes given the emerging nature of the evidence base, and for smokeless tobacco for which there is limited research on its use as a potential a harm reduction product [104]. It is somewhat surprising in the case of NRT, however, given its relevance to harm reduction strategies, especially in the UK. While there is extensive evidence on the equity impacts of cessation support *including* NRT, we found no academic literature on the equity impacts of NRT in isolation from other forms of cessation support. One study [101] came close, but did not provide sufficient detail for us to examine the effects of NRT alone.

Similar to Hartwell et al. [15] we found studies assessing e-cigarette ‘ever-use’ were more likely to suggest a negative equity impact. However, diverting from Hartwell et al’s study we found potentially positive outcomes for e-cigarette current use, although this pattern is not consistent. Thus there is some evidence to suggest that an initial socioeconomic gradient (with higher use in high SES groups) may flatten over time.

Studies of smokeless tobacco use by SES provide a possible indication of how e-cigarette use may be distributed once this has become as established part of the nicotine consumption landscape. Our review found a clear pattern in smokeless tobacco consumption by SES, with consistently higher current and ever use among low SES groups. While this pattern is consistent with a theoretically equity-positive distribution (Fig. 2) combustible tobacco use remains higher among lower SES groups. In other words, we can speculate that smokeless tobacco tends to be used in combination with combustible tobacco (a form of dual use), and thus does not help reduce tobacco use in lower SES groups. This provides a useful perspective on the likely trajectory of e-cigarette consumption, currently at the ‘early adopter’ stage of the diffusion of innovations theory [16] with higher use among high SES groups (the typical ‘early adopters’). While this theory suggests that – over time – e-cigarette use is likely to become more equitable by SES, the experience of countries with mature smokeless tobacco markets (the US, Norway and Sweden) suggests that e-cigarette use is likely co-exist with conventional tobacco use rather than displacing it within low SES groups.

We recognise there are some challenges in comparing patterns of use among the three types of NCNP covered in this review, which have significantly different histories of regulation and harm reduction appeal. However, our main comparison has been between e-cigarettes and smokeless tobacco, both of which straddle positions of therapeutic and recreational use: as harm reduction products and as part of existing and developing cultural norms.

Regardless of the availability or effectiveness of NCNPs for smoking cessation, a common feature of quit attempts is their lower success among low SES smokers [4, 105]. Qualitative studies point to the social, economic and cultural circumstances that encourage nicotine dependence and act as a barrier to cessation [28, 106–108]. Therefore, we would caution against undue optimism regarding the impact of any NCNP on smoking inequalities, and tend to agree with Thirlway’s [109] assessment that e-cigarettes are likely to complicate rather than transform these inequalities.

### Limitations

Our review has some limitations, most notably the lack of available research – meaning our findings and conclusions are necessarily based on indirect evidence of the equity impacts of e-cigarettes and other NCNPs.

The available evidence is further limited by challenges in assessing NCNP use and SES [110]. Studies used a range of definitions for NCNP use, which may not always be comparable; furthermore, surveys of e-cigarette use did not distinguish between e-liquid with and without nicotine. In order to synthesise evidence from a range of studies, we necessarily treated SES indicators as equivalent although the categorisation and meaning of education- and income-based measures may differ across study contexts. Another important limitation of this review is that we have been obliged to combine findings from countries with diverse regulation, availability and cultural norms regarding e-cigarettes, smokeless tobacco and NRT. Contextual factors such as these may influence the use of these products in ways that are relevant to their distribution by SES, but an assessment of such effects is beyond the scope of this review (and unfeasible given limitations in the available evidence). This limitation may be particularly relevant in the case of evidence on smokeless tobacco use, where 13 of the 16 ‘best evidence’ studies came from the USA. It has been suggested that the US has a distinctive pattern of smokeless tobacco use, and that – as more ‘mature’ markets - Sweden and Norway might provide a more robust indication of long-term patterns of dual smokeless tobacco and smoked tobacco use [111]; in future reviews, it would therefore be helpful to have more evidence on the distribution of smokeless tobacco use by SES from these countries.

We also note that, in studies using more than one indicator of SES (e.g. education *and* income), multivariate regression analyses were sometimes adjusted for the other indicator, which may mask the true magnitude of the socioeconomic gradient in product use [112]. This limitation applies only to the lowest tier of our ‘best evidence’ gradient, i.e. tier 3 studies (see Additional file 4). Since tier 3 studies using more than one SES indicator comprise less than a quarter of the ‘best evidence’ studies for adults, and none for young people (see Additional file 6), the overall effect on our findings is likely to be modest.

We decided not to include grey literature or studies not in English in this review, given the difficulty of identifying and appraising all relevant material. When so much of the academic literature is of limited value to an equity-focused review, the benefit of incorporating a broader range of evidence must be weighed against the limited value of much of the evidence identified. Our decision to undertake a ‘best evidence’ review reflects this trade-off.

Finally, the evidence base on e-cigarettes is rapidly evolving, and our review necessarily includes only evidence published at the time the search was undertaken. Nonetheless, our comparison of three NCNPs, our focus on countries at stage IV of the cigarette epidemic, and our comprehensive quality appraisal to identify best evidence situate this study as a key contribution in public health’s evolving understanding of the impacts of NCNPs on smoking inequalities.

## Conclusion

Our review compares the potential equity impact of three NCNPs at different stages of market maturity. The findings indicate a consistent pattern only in relation to the most established product, smokeless tobacco, where use is higher among low SES groups. While this suggests the potential for reducing SES inequalities in use of smoked tobacco, evidence shows that such a reduction has not occurred with low SES groups tending to have higher use of both smokeless tobacco and smoked tobacco (rather than smokeless tobacco use displacing consumption of smoked tobacco). E-cigarettes are a more recent commercial product and current patterns suggest their use is at an earlier stage of diffusion throughout society [16]; like smokeless tobacco, however, their credentials as harm reduction or cessation products remains contested. Our findings therefore raise the possibility that e-cigarettes may follow the same path as smokeless tobacco: as a common susceptibility rather than substitution for smoking among low SES groups. This prospect may reflect the possible intentions of the tobacco industry to establish vaping as something to be used alongside smoking, rather than a direct replacement [113].

This finding should be an important consideration of healthcare providers and policy makers who might wish to include e-cigarettes as part of harm reduction strategies. It will be important to continue monitoring the use and impacts of e-cigarettes in relation to SES and other markers of social position in order to maximise the devices’ potential to reduce inequalities in smoking while minimising their potential to have the opposite effect.

## Supplementary information

**Additional file 1.** PRISMA-E Checklist https://documentcloud.adobe.com/link/track?uri=urn%3Aaaid%3Ascds%3AUS%3A8e22ee52-cf84-4216-a119-192a5e92bfc1

**Additional file 2.** Example of search terms https://documentcloud.adobe.com/link/track?uri=urn%3Aaaid%3Ascds%3AUS%3A9b50af08-7a86-43a2-ab33-b9e15ed2e379

**Additional file 3.** Data Extraction and quality appraisal template https://documentcloud.adobe.com/link/track?uri=urn%3Aaaid%3Ascds%3AUS%3Ac6d7db29-6f62-4b30-baa3-ce6b29d6cf02

**Additional file 4.** Relevance of study findings table https://documentcloud.adobe.com/link/track?uri=urn%3Aaaid%3Ascds%3AUS%3A9c76eb56-cfa4-4653-87a9-9ea33b9b186d

**Additional file 5.** Quality appraisal appendix https://documentcloud.adobe.com/link/track?uri=urn%3Aaaid%3Ascds%3AUS%3A367c5bca-58a8-4aa8-af45-aa1ea5944077

**Additional file 6.** Potential equity outcome tables https://documentcloud.adobe.com/link/track?uri=urn%3Aaaid%3Ascds%3AUS%3A3843ad16-0748-4561-993e-bf93b3208c9a

**Additional file 7.** Full list of studies included in review https://documentcloud.adobe.com/link/track?uri=urn%3Aaaid%3Ascds%3AUS%3Ac6a4acca-8e43-4e7e-a5ad-cff8464de9b8

## Abbreviations

NCNP: Non-combustible nicotine-containing products
NRT: Nicotine Replacement Therapy
PRISMA: Preferred Reporting Items for Systematic Reviews and Meta-Analyses
SES: Socioeconomic Status
