# Supplementary material for: Potential for non-combustible nicotine products to reduce socioeconomic inequalities in smoking: a systematic review and synthesis of best available evidence
Source: BMC Public Health. 2019 Nov 6;19:1469. doi: 10.1186/s12889-019-7836-4 (PMC6836524; doi:10.1186/s12889-019-7836-4)
Supplement: Supplementary file 2 — Additional file 2. Example of search terms https://documentcloud.adobe.com/link/track?uri=urn%3Aaaid%3Ascds%3AUS%3A9b50af08-7a86-43a2-ab33-b9e15ed2e379 [file 12889_2019_7836_MOESM2_ESM.pdf]

Additional File 2: Example of search terms

Paper: Potential for non-combustible nicotine products to reduce socioeconomic inequalities in smoking: a systematic review and synthesis of best available evidence.
Authors: Mark Lucherini, Sarah Hill, Katherine Smith

Search terms applied in MEDLINE

<u>Smoking/tobacco/nicotine terms</u>

smoking
tobacco
"tobacco use disorder"
nicotine
smoker OR smokers
cigar*

<u>Non-combustible Nicotine Product Terms</u>

smokeless
(smokeless or smoke-less or "smoke less") AND tobacco
e-cig* OR "electronic cig*" OR electronic-cig*
ENDS OR "electronic nicotine delivery system*"
ANDS OR "alternative nicotine delivery system*"
snuff
snus
"nicotine replacement therap*" OR NRT
"nicotine replacement treatment*"
"nicotine replacement intervention*"
"heat not burn" OR "heat-not-burn"
vaporiser* OR vapouriser* OR vaporizer* OR vapourizer*
vape* OR vaping
(shop OR store) AND (vape* OR vapour OR vapor)
e-hookah* OR "electronic hookah*" OR electronic-hookah*
e-shisha* OR "electronic shisha*" OR electronic-shisha*
(nicotine OR tobacco) AND (patch* OR gum* OR inhaler* OR inhalator* OR lozenge* OR strip* OR stick* OR tablet* OR "nasal spray*" OR "mouth spray*" OR dissolvable OR oral OR dip* OR chew*)

<u>Inequality terms</u>

socioeconomic OR "socio economic" OR socio-economic
inequal*
disparit*
depriv*
disadvantage*
educat*
social AND (class* OR group* OR grade* OR context* OR status)
unemploy*

income
poverty
SES
demographic*
poor
equity
social AND (disadvant* OR exclusion OR excluded OR depriv*)

(Medline specific)
exp socioeconomic factors/
exp public assistance/
exp social welfare/
exp vulnerable populations/
