# Supplementary material for: Potential for non-combustible nicotine products to reduce socioeconomic inequalities in smoking: a systematic review and synthesis of best available evidence
Source: BMC Public Health. 2019 Nov 6;19:1469. doi: 10.1186/s12889-019-7836-4 (PMC6836524; doi:10.1186/s12889-019-7836-4)
Supplement: Supplementary file 3 — Additional file 3. Data Extraction and quality appraisal template https://documentcloud.adobe.com/link/track?uri=urn%3Aaaid%3Ascds%3AUS%3Ac6d7db29-6f62-4b30-baa3-ce6b29d6cf02 [file 12889_2019_7836_MOESM3_ESM.pdf]

Additional File 3. Data extraction and quality appraisal template

Paper: Potential for non-combustible nicotine products to reduce socioeconomic inequalities in smoking: a systematic review and synthesis of best available evidence.
Authors: Mark Lucherini, Sarah Hill, Katherine Smith

Bibliographic Details

……………………………………………………………….

Original research aim of study

…………………………………………………

Why study is relevant to our review (i.e. how does the study provide evidence of differences (or lack of difference) in use or impact of NCNP by SES?)

…………………………………………………

Study details

Study design	
Study location	
Participant characteristics	
Study period	
Study size	
Type of product	
Indicators(s) of SES	

What NCNP is being examined in study?

**Product**	**Mark 'X' if relevant**	**Notes**
E-cigarettes		
NRT		
Smokeless tobacco: snus		
Smokeless tobacco: other		

How is the NCNP being examined by SES?

**Study outcome: product use & SES**	**Mark 'X' if covered**	**Notes (current use, ever use etc)**
Prevalence of use		
Prevalence of NCNP use among smokers by SES		
Distribution of user groups by SES		
Association between SES and NCNP (no prevalence)		

Main findings of relevance for our study

INDIRECT EVIDENCE (PRODUCT USE): What do the study findings tell us about the use of NCNP by SES?

....

	Low SES > High SES	Low SES = High SES	Low SES < High SES	Unclear
Ever use				
Current use				

What do the study findings tell us about the use of conventional tobacco by SES in this population?

....

	Low SES > High SES	Low SES = High SES	Low SES < High SES	Unclear	No data
Tobacco product					

What do the study findings tell us about dual use (ie NCNP + conventional tobacco) by SES in this population?

....

	Low SES > High SES	Low SES = High SES	Low SES < High SES	Unclear	No data
Dual use (define)					

Anything other information about conventional tobacco use (by SES) in this population (text)?

....

Critical Appraisal

	Quality appraisal question	Evaluation	Notes
1	Does the research question/aim include a primary focus on use/impacts of a NCNP by SES?		
2	Does the methodology allow us to assess use/impacts of a NCNP by SES?		
3	Was the recruitment strategy appropriate? (including sample selection, response rates and follow-up, where relevant)		

4	Did the study include appropriate indicators of SES?		
5	Did the study include a relevant measure of NCNP use or impact?		
6	Were the methods of data analysis appropriate?		
7	Is the study adequately powered for SES comparisons?		
8	Have the authors taken into account any potential confounding factors, including age?		
9	Can the findings (in relation to harm reduction patterns by SES) be generalised, and – if so – to what level?		

SUMMARY

What might these results suggest about equity outcomes (indicate possible positive, negative, neutral or unclear outcomes)? Overall quality classification in relation to our research question (Low, Medium, High)

……………………………………………

Evidence tier qualification

……………………………………………
