# Supplementary material for: Potential for non-combustible nicotine products to reduce socioeconomic inequalities in smoking: a systematic review and synthesis of best available evidence
Source: BMC Public Health. 2019 Nov 6;19:1469. doi: 10.1186/s12889-019-7836-4 (PMC6836524; doi:10.1186/s12889-019-7836-4)
Supplement: Supplementary file 4 — Additional file 4. Relevance of study findings table https://documentcloud.adobe.com/link/track?uri=urn%3Aaaid%3Ascds%3AUS%3A9c76eb56-cfa4-4653-87a9-9ea33b9b186d [file 12889_2019_7836_MOESM4_ESM.pdf]

Additional File 4. Relevance of study findings table

Paper: Potential for non-combustible nicotine products to reduce socioeconomic inequalities in smoking: a systematic review and synthesis of best available evidence.
Authors: Mark Lucherini, Sarah Hill, Katherine Smith

Tier	Description	Specific criteria
Tier 1	**Evidence of NCNP use (prevalence) by SES,** with adequate power to detect differences by SES<br>**AND**<br>**information on cigarette use (prevalence) by SES in the same population**	Cross-sectional data (including from cohort studies) demonstrating:<br>- Prevalence of NCNP use by SES<br>- Statistically significant difference in NCNP use by SES, or sufficiently powered to confirm lack of difference<br>- Prevalence of cigarette use by SES
Tier 2	**Evidence of NCNP use (prevalence) by SES,** with adequate power to detect differences by SES,<br>but no information on cigarette use by SES in the same population	Cross-sectional data (including from cohort studies) demonstrating:<br>- Prevalence of NCNP use by SES<br>- Statistically significant difference in NCNP use by SES, or sufficiently powered to confirm lack of difference
Tier 3	**Indirect evidence of NCNP and cigarette use in relation to SES, but no estimates of use (prevalence) in the underlying population**	Cross-sectional data (including from cohort studies) demonstrating:<br>- Prevalence of NCNP use by SES among smokers, *OR*<br>- Association between SES and NCNP/tobacco use (Odds Ratios only), *OR*<br>- Distribution of different product user groups (smokers, e-cig users, dual users) by SES
Tier 4	Evidence of pattern of NCNP by SES, but no test of statistical significance / no evidence of adequate study power	Cross-sectional data (including from cohort studies) demonstrating:<br>- Prevalence of NCNP use by SES, inadequately powered

Best evidence adults include Tier 1, 2 and 3 studies of medium and higher quality.

Best evidence for young people includes only Tier 1 studies of high or medium quality (ie studies must include evidence on tobacco use by SES in the same population).
