# Supplementary material for: Potential for non-combustible nicotine products to reduce socioeconomic inequalities in smoking: a systematic review and synthesis of best available evidence
Source: BMC Public Health. 2019 Nov 6;19:1469. doi: 10.1186/s12889-019-7836-4 (PMC6836524; doi:10.1186/s12889-019-7836-4)
Supplement: Supplementary file 5 — Additional file 5. Quality appraisal appendix https://documentcloud.adobe.com/link/track?uri=urn%3Aaaid%3Ascds%3AUS%3A367c5bca-58a8-4aa8-af45-aa1ea5944077 [file 12889_2019_7836_MOESM5_ESM.pdf]

Additional File 5. Quality appraisal appendix

Paper: Potential for non-combustible nicotine products to reduce socioeconomic inequalities in smoking: a systematic review and synthesis of best available evidence.

Authors: Mark Lucherini, Sarah Hill, Katherine Smith

**Study**	**Tobacco products by SES?***	**Recruitment?****	**Appropriate indicators of SES?+**	**NCNP use?++**	**Data analysis appropriate?+++**	**Adequate study power? **++**	**Confounding factors taken into account?*****	**Generalisability ±**	**Quality appraisal**
Acortt et al 2005	No	Yes	Yes	Partially	Yes	Partially	Yes	National	Medium
Agaku et al 2014	Yes	Partially	Yes	Yes	Partially	Partially	No	National	Low
Alberg et al 2005	No	Partially	Yes	Partially	Partially	No	No	Regional, subpopulation	Low
*Babineau et al 2015	No	Yes	Partially	Yes	Yes	Partially	Yes	National, subpopulation	Low
Backinger et al 2008	No	Yes	Yes	Partially	Partially	Unclear	No	National, subpopulation	Low
**Bhattacharyya 2012	No	Yes	Unclear	Yes	Yes	Unclear	Unclear	National	Low
Beiner et al 2011	No	Yes	Partially	Partially	Yes	Partially	Partially	National	Medium
Brown et al 2014	Yes	Partially	Partially	Yes	Yes	Yes	Yes	Unclear	Medium

Study	Tobacco products by SES?*	Recruitment?**	Appropriate indicators of SES?+	NCNP use?++	Data analysis appropriate?+++	Adequate study power? **++	Confounding factors taken into account?***	Generalisability ±	Quality appraisal
Carrieri and Jones 2016	Yes	Yes	Yes	Partially	Yes	Yes	Yes	National	Medium
Chang et al 2016	No	Yes	Yes	Yes	Yes	Yes	Yes	National, Subpopulation	Medium
**Chivers et al 2016	Yes	Partially	Yes	No	Yes	Partially	Yes	Not generalisable	Low
Christensen et al 2014	Yes	Yes	Yes	Yes	Partially	Unclear	Partially	Not generalisable	Low
Clare et al 2014	Yes	Yes	Yes	Partially	Yes	Yes	Yes	National	Medium
**Douptcheva et al 2013	Yes	Partially	Partially	Partially	Partially	Unclear	Unclear	Not generalisable	Low
Engstrom et al (2010)	Yes	Yes	Yes	Yes	Yes	Partially	Yes	National	Medium
*Fischer et al 2014	No	Partially	Partially	Yes	Yes	Unclear	Yes	Subpopulation	Low
Gallus et al 2014	Yes	Yes	Partially	Yes	No	No	Yes	Not generalisable	Low
*Grotvedt et al 2008	Yes	Yes	Yes	Yes	Yes	Yes	Yes	National, Subpopulation	Medium
**Hill et al 1992	No	Yes	Partially	Yes	Yes	Partially	Unclear	Regional, Subpopulation	Low

**Study**	**Tobacco products by SES?***	**Recruitment?****	**Appropriate indicators of SES?+**	**NCNP use?++**	**Data analysis appropriate?+++**	**Adequate study power? **++**	**Confounding factors taken into account?*****	**Generalisability ±**	**Quality appraisal**
Hu et al 2016	Yes	Partially	Yes	Yes	Yes	Yes	No	National	Medium
Huang et al 2016	Yes	Yes	Yes	Yes	Yes	Yes	Yes	National	High
*Khoury et al 2016	No	Yes	Yes	Yes	Unclear	Unclear	Yes	Regional	Low
**King et al 2013	No	Yes	Yes	Partially	Partially	Partially	Unclear	National	Low
King et al 2015	No	Yes	Yes	Yes	Yes	No	Yes	National	Low
*Kinnunen et al 2016	No	Partially	Yes	Yes	Yes	Yes	Yes	Subpopulation	Medium
Kotz and West 2009	No	Yes	Yes	Yes	Partially	Yes	No	National	Medium
Kushnir et al 2017	Yes	Partially	Partially	Yes	Partially	Unclear	No	National	Low
Kvaavik et al 2016	Yes	Partially	Yes	Yes	Yes	Yes	Yes	National, subpopulation	Medium
McMillen et al 2012	No	Yes	Yes	Partially	Partially	No	No	National	Low
Mazurek et al 2014	Yes	Yes	Yes	Yes	Yes	Yes	Unclear	Subpopulation	Medium
Mushtaq et al 2012	Yes	Yes	Yes	Yes	Yes	Partially	Yes	National	Medium

**Study**	**Tobacco products by SES?***	**Recruitment?****	**Appropriate indicators of SES?+**	**NCNP use?++**	**Data analysis appropriate?+++**	**Adequate study power? **++**	**Confounding factors taken into account?*****	**Generalisability ±**	**Quality appraisal**
Nelson et al 1996	No	Yes	Yes	Yes	Yes	No	Yes	Regional/Subpopulation	Low
Norberg et al 2011	No	Partially	Yes	Partially	Yes	Yes	Yes	Regional, subpopulation	Medium
Novotny et al 1989	No	Yes	Yes	Yes	Yes	Partially	Unclear	National, Subpopulation	Medium
*Overland et al 2010	Yes	Yes	Yes	Yes	Yes	Partially	Yes	National, Subpopulation	Medium
Pearson et al 2012	Yes	Yes	Yes	Partially	Yes	Yes	Yes	National	Medium
*Pedersen and Von Soest 2014	Yes	Yes	Yes	Yes	Partially	No	Yes	National, Subpopulation	Low
*Pedersen and Bakken 2016	Yes	Yes	Yes	Yes	Yes	Yes	Yes	Regional, subpopulation	High
Regan et al 2013	No	Partially	Yes	Yes	Yes	Yes	No	National	Low
*Rennie et al 2016	Yes	Partially	Partially	Partially	Yes	Yes	Yes	Regional/Subpopulation	Medium
Roberts et al 2016	Yes	Yes	Yes	Partially	No	No	No	National	Low

Study	Tobacco products by SES?*	Recruitment?**	Appropriate indicators of SES?+	NCNP use?++	Data analysis appropriate?+++	Adequate study power? **++	Confounding factors taken into account?***	Generalisability ±	Quality appraisal
Rodu and Cole 2009	No	Unlcear	Yes	Yes	Partially	Partially	Unclear	National	Medium
Romito and Saxton 2014	No	Partially	Partially	Yes	No	No	Unclear	Not generalisable	Low
Syamlal et al 2016 (EC)	No	Partially	Yes	Yes	Yes	Yes	Yes	Subpopulation	Medium
Syamlal et al 2016 (SLT)	Yes	Yes	Yes	Partially	Yes	Yes	Unclear	National	Medium
Sung et al 2016	Yes	Partially	Yes	Yes	Yes	Yes	Yes	National	Medium
Thorne et al 2010	Yes	Yes	Yes	Yes	Yes	Partially	Yes	National	Medium
Vijayaraghavan et al 2014	Yes	Yes	Yes	Partially	Yes	Yes	Yes	National	Medium
*Wang et al 1994	Yes	Yes	Unclear	Yes	Partially	Unclear	No	National, Sub population	Low
*Wang et al 1998	Yes	Yes	Partially	Yes	Partially	Partially	Unclear	National, Subpopulation	Low
Weaver et al 2015	Yes	Yes	Yes	Yes	Partially	Partially	No	National	Low

Study	Tobacco products by SES?*	Recruitment?**	Appropriate indicators of SES?+	NCNP use?++	Data analysis appropriate?+++	Adequate study power? **++	Confounding factors taken into account?***	Generalisability ±	Quality appraisal
White et al 2016	Yes	Yes	Yes	Yes	Yes	Yes	Yes	National	High
Wilson and Wang 2016	No	Yes	Yes	Yes	Yes	Partially	Yes	National	Medium
*Wills et al 2016	Yes	Yes	Yes	Yes	Yes	Yes	Yes	Subpopulation	Medium

<u>Quality Appraisal Guide</u>

* Does the methodology allow us to assess use of combustible tobacco products (cigarettes) by SES?
** Was the recruitment strategy appropriate?
+ Did the study include appropriate indicators of SES?
++ Did the study include a relevant measure of NCNP use or impact?
+++ Were the methods of data analysis appropriate?
**++ Was the study adequately powered to assess differences by SES?
*** Have the authors taken into account any potential confounding factors, including age?
± Can the findings be generalised, and – if so – to what level?

<u>Studies to be categorised as Medium if any:</u>
Appropriate indicators of SES = Partially (in most cases due to binary measure, ie high income vs low income)
AND/OR
NCNP use = Partially (in most cases due to ever use being only measure)
AND/OR
Confounding factors taken into account = Partially (in most cases because age has not been taken into consideration)
AND/OR

Recruitment = Partially (in most cases due low response rate, high attrition, or problems with sample selection)
AND/OR
Data analysis = Partially
AND/OR
Study power = Partially (is study adequately powered to assess differences by SES)
AND/OR
Generalisability = Partially (ie subpopulation)

Studies to be categorised as Low if any:
Appropriate indicators of SES = No (in most cases due to binary measure, ie high income vs low income)
AND/OR
NCNP use = No (in most cases due to ever use being only measure)
AND/OR
Confounding factors taken into account = No (in most cases because age has not been taken into consideration)
AND/OR
Recruitment = No (in most cases due low response rate, high attrition, or problems with sample selection)
AND/OR
Data analysis = No
AND/OR
Study power = Partially (is study adequately powered to assess differences by SES)
AND/OR
Generalisability = No (not generalisable)

Factors only indicative of quality for studies on young people:
Tobacco product by SES = No (study offers no data for combustible tobacco use among sample by SES or offers only dual use data with no baseline characteristics)

Notes

* Due to the nature of tobacco use patterns among young people being unpredictable many of the studies on young people were marked lower in quality than the rating criteria above might suggest.

** In some cases the number of partially or unclear markers has led to study quality to be assessed as Low.
