# Supplementary material for: Potential for non-combustible nicotine products to reduce socioeconomic inequalities in smoking: a systematic review and synthesis of best available evidence
Source: BMC Public Health. 2019 Nov 6;19:1469. doi: 10.1186/s12889-019-7836-4 (PMC6836524; doi:10.1186/s12889-019-7836-4)
Supplement: Supplementary file 6 — Additional file 6. Potential equity outcome tables https://documentcloud.adobe.com/link/track?uri=urn%3Aaaid%3Ascds%3AUS%3A3843ad16-0748-4561-993e-bf93b3208c9a [file 12889_2019_7836_MOESM6_ESM.pdf]

Additional File 6. Potential equity outcome tables

Paper: Potential for non-combustible nicotine products to reduce socioeconomic inequalities in smoking: a systematic review and synthesis of best available evidence.
Authors: Mark Lucherini, Sarah Hill, Katherine Smith

Tables:

- Table 1: Studies included in adult e-cigarette best evidence synthesis
- Table 2: All other adult e-cigarettes studies (not included in adults best evidence synthesis)
- Table 3: Young people and e-cigarette studies
- Table 4: Studies included in adult smokeless tobacco best evidence synthesis
- Table 5: All other adult SLT studies (not included in adults best evidence synthesis)
- Table 6: Young people and smokeless tobacco studies
- Table 7: All nicotine replacement therapy studies

Table 1: Studies included in adult e-cigarette best evidence synthesis

Study	Data collection	Location	Study size	Measure(s) of SES	Main findings by SES measure(s) for EC**	Potential impact on smoking inequalities - Current Use	Potential impact on smoking inequalities - Ever Use	Evidence Tier	Quality Appraisal
Brown et al. 2014	2012	UK, England	3538	Occupational group	high > low	Negative	None	3	M
Carrieri and Jones 2016	2013	UK, England	1697	EDU, INC	INC high > low<br>EDU high > low	None	Negative (EDU, INC)	3	M

Study	Data collection	Location	Study size	Measure(s) of SES	Main findings by SES measure(s) for EC**	Potential impact on smoking inequalities - Current Use	Potential impact on smoking inequalities - Ever Use	Evidence Tier	Quality Appraisal
Huang et al. 2016	2013	US	17522	EDU, INC	EDU low > high (CU). EDU unclear (EU) INC unclear (CU, EU).	Unclear (INC) Positive (EDU)	Unclear (EDU, INC)	1	H
Hu et al. 2016 (also included in table 4)	2013-2014	US	26437	EDU, INC	EDU unclear INC low > high	Positive (INC) Unclear (EDU)	None	1	M
Pearson et al. 2012	2010	US	1308 3239*	EDU	EDU high > low	None	Negative	3	M
Syamlal et al. 2016	2014	US	146342	EDU, INC	INC low > high EDU low = high	Positive (INC) Neutral (EDU)	None	2	M
Wilson and Wang 2016	2014	US	34356	EDU, INC	EDU unclear (CU) INC unclear (CU) EDU unclear (EU) INC high > low (EU)	Unclear (EDU, INC)	Unclear (EDU) Negative (INC)	3	M

*Two samples considered separately but use following similar gradient for each.
**EC, e-cigarettes; EDU, education; INC, income; CU, current use; EU, ever use

Table 2: All other adult e-cigarettes studies (not included in adults best evidence synthesis)

Study	Data collection	Location	Study size	Measure(s) of SES	Main findings by SES measure(s) for EC**	Potential impact on smoking inequalities - Current use	Potential impact on smoking inequalities - Ever Use	Evidence Tier	Quality Appraisal
Agaku et al. 2014 (also included in table 5)	2012-2013	US	27026	EDU, INC	EDU low = high<br>INC low = high	Unclear (EDU, INC, EC)	None	4	L
Chivers et al 2016	2014	US	800	EDU	EDU low > high	Positive	None	3	L
Christensen et al 2014	2012-2013	US, Kansas	9656	EDU, INC	INC high > low<br>EDU low > high	None	Negative (INC)<br>Positive (EDU)	3	L
Douptcheva et al 2013	2010-2013	Switzerland	5081	EDU	EDU low > high	None	Positive	4	L
Gallus et al. 2014	2013	Italy	3000	EDU	EDU unclear	None	Unclear	4	L

Study	Data collection	Location	Study size	Measure(s) of SES	Main findings by SES measure(s) for EC**	Potential impact on smoking inequalities - Current use	Potential impact on smoking inequalities - Ever Use	Evidence Tier	Quality Appraisal
King et al. 2013	2010<br>2011	US	4050<br>4184<br>2125*	EDU, INC	EDU unclear<br>INC unclear	None	Unclear (EDU, INC)	4	L
King et al. 2015	2010<br>2011<br>2012<br>2013	US	2,502<br>4,050<br>4,170<br>4,033*	EDU, INC	EDU low = high (CU)<br>INC unclear (CU)<br>EDU unclear (EU)<br>INC low = high (EU)	Neutral (EDU)<br>Unclear (INC)	Unclear (EDU)<br>Neutral (INC)	4	M
McMillen et al. 2012 (also included in table 5)	2010	US	3240	EDU	EDU unclear	None	Unclear	4	L
Regan et al. 2013	2010	US	10328	EDU, INC	EDU low > high (CU, EU)<br>INC low > high (EU)<br>INC low = high (CU)	Positive (EDU)<br>Neutral (INC)	Positive (EDU, INC)	2	L
Weaver et al. 2016	2014	US	5717	EDU, INC	INC low > high (CU, EU)<br>EDU low = high (CU, EU)	Positive (INC)<br>Neutral (EDU)	Positive (INC)<br>Neutral (EDU)	1	L

*Each sample considered separately but use following similar gradient for each.
**EC, e-cigarettes; EDU, education; INC, income; CU, current use; EU, ever use

Table 3: Young people and e-cigarette studies

Authors	Data collection	Location	Study size	Measure(s) of SES	Main findings by SES measure(s) for EC*	Main findings for combustible tobacco use (general SES)	Potential impact on smoking inequalities - Current Use	Potential impact on smoking inequalities - Ever Use	Evidence Tier	Critical Appraisal
Babineau et al. 2016	2014	Ireland	821	Neighbourhood deprivation	low deprivation > high (EU)<br>unclear (CU)	No data	Positive	Unclear	3	L
Khoury et al. 2016	203-2014	Canada Niagara region	2367	INC	INC low > high	No data	None	Positive	3	L
Kinnunen et al. 2016	2013 2015	Finland	13369 (pooled)	EDU	EDU low > high (EU)<br>EDU unclear (CU)	No data	Positive	Unclear	2	M
Rennie et al. 2016	2014-2015	France, Hauts-de-Seine	1486	Composite index	high > low	High > low	None	Unclear	3	M
Wills et al. 2016	2013-2014	US, Hawaii	2338	EDU	EDU low > high	Low = high	Unclear	None	3	M

*EC, e-cigarette; EDU, education; INC, income; CU, current use; EU, ever use

Table 4: Studies included in adult smokeless tobacco best evidence synthesis

Authors	Data collection	Location	Study size	Measure(s) of SES	Main findings by SES measure(s) for SLT**	Potential impact on smoking inequalities - Current Use	Potential impact on smoking inequalities - Ever use	Evidence Tier	Critical Appraisal
Accortt et al. 2005	1971-1975	US	12172	EDU, INC	EDU low > high<br>INC low > high	None	Positive (EDU, INC)	2	M
Biener et al. 2011	2008	US	4067	EDU	EDU low > high	None	Positive	3	M
Chang et al 2016	1992-2011	US	Unclear	EDU, INC	EDU low > high<br>INC low = high	Positive (EDU)<br>Neutral (INC)	None	2	M
Engstrom et al. 2010	2006	Sweden, Stockholm County	34707	EDU, INC,	EDU low > high<br>INC unclear	Positive (EDU)<br>Unclear (INC)	None	3	M
Hu et al. 2016 (also included in table 1)	2013-2014	US	26437	EDU, INC	EDU low > high<br>INC low = high	Positive (EDU)<br>Neutral (INC)	None	1	M
Kvaavik et al. 2016	2011-2012	Norway	13756	EDU	EDU low > high	None	Positive	3	M
Mazurek et al. 2014	2005<br>2010	US	19445<br>15649	EDU, INC	EDU low > high<br>INC low = high	Neutral (INC)<br>Positive (EDU)	None	1	M

Authors	Data collection	Location	Study size	Measure(s) of SES	Main findings by SES measure(s) for SLT**	Potential impact on smoking inequalities - Current Use	Potential impact on smoking inequalities - Ever use	Evidence Tier	Critical Appraisal
Mushtaq et al. 2012	2010	US	169487	EDU, INC	EDU low > high<br>INC low > high	Positive (INC, EDU)	None	1	M
Norberg et al. 2011	1990-2006	Sweden, Vasterbotten County	92563	EDU, INC	INC low = high<br>EDU unclear	None	Neutral (INC)<br>Unclear (EDU)	2	M
Novotny et al. 1989	1986	US	6377	INC, EDU	EDU low > high<br>INC low > high	Positive (EDU, INC)	Positive (EDU, INC)	2	M
Rodu and Cole. 2009	2005	US	103709	EDU, INC	EDU low > high<br>INC unclear	Positive (EDU)<br>Neutral (INC)	None	2	M
Syamlal et al. 2016	2012-2014	US	61402	EDU, INC	EDU low > high<br>INC unclear	Positive (EDU)<br>Unclear (INC)	None	1	M
Sung et al. 2016	1998 2000 2005 2010 (pooled data)	US	117816	EDU, INC	EDU low > high<br>INC low = high	Positive (EDU)<br>Neutral (INC)	None	1	M
Thorne et al 2010	2008	US, 13 states across country	~400349	EDU, INC	EDU low > high<br>INC low = high	Positive (EDU)<br>Neutral (INC)	None	1	M
Vijayaraghavan et al. 2014	2006-2012	US	54239	EDU, INC	EDU low > high<br>INC unclear	None	Unclear (INC)<br>Positive (EDU)	3	M

Authors	Data collection	Location	Study size	Measure(s) of SES	Main findings by SES measure(s) for SLT**	Potential impact on smoking inequalities - Current Use	Potential impact on smoking inequalities - Ever use	Evidence Tier	Critical Appraisal
White et al. 2016	2012	US	37869	EDU, INC	EDU low > high<br>INC low = high	Positive (EDU)<br>Neutral (INC)	None	3	H

*Each sample considered separately but use following similar gradient for each.
**SLT, smokeless tobacco; EDU, education; INC, income; CU, current use; EU, ever use

Table 5: All other adult smokeless tobacco studies (not included in adults best evidence synthesis)

Authors	Data collection	Location	Study size	Measure(s) of SES	Main findings by SES measure(s) for SLT*	Potential impact on smoking inequalities - Current Use	Potential impact on smoking inequalities - Ever Use	Evidence Tier	Critical Appraisal
Agaku et al. 2014 (also included in table 2)	2012-2013	US	27026	EDU, INC	EDU low = high<br>INC low = high	Neutral (EDU, INC)	None	4	L
Backinger et al. 2008	1995/95<br>1998<br>2000<br>2001/02	US	43232<br>134481<br>26216<br>37210**	EDU, INC	EDU low > high<br>INC low = high	None	Positive (EDU)<br>Neutral (INC)	3	L
Bhattacharyya. 2012	2000<br>2005<br>2010	US	86270 (pooled)	EDU, INC	EDU low > high<br>INC unclear	Positive (EDU)<br>Unclear (INC)	None	3	L
McMillen et al. 2012 (also	2010	US	3240	EDU	EDU low = high	None	Neutral	4	L

Authors	Data collection	Location	Study size	Measure(s) of SES	Main findings by SES measure(s) for SLT*	Potential impact on smoking inequalities - Current Use	Potential impact on smoking inequalities - Ever Use	Evidence Tier	Critical Appraisal
included in table 2)									
Nelson et al. 1996	1992-1993	US, Iowa, Montana, West Virginia, Indiana	1853 1549 1007 1826*	EDU	EDU low > high	Positive	None	4	L
Roberts et al. 2016	2013-2013	US	136147	INC	INC unclear	Unclear	None	4	L
Romito and Saxton. 2014	2010	US, Indiana	472	EDU	EDU unclear	None	Unlcear	2	L

*SLT, smokeless tobacco; EDU, education; INC, income; CU, current use; EU, ever use

**Sample considered separately in analysis in paper but all followed same pattern for prevalence of use

Table 6: Young people and smokeless tobacco studies

Authors	Data collection	Location	Participants	Study size	Measure(s) of SES	Main findings by SES measure(s) for SLT***	Main findings for combustible tobacco use (general SES)	Potential impact on smoking inequalities - Current Use	Potential impact on smoking inequalities - Ever Use	Evidence Tier	Critical Appraisal
Hill et al1992	Unclear	US, North Carolina	10-14 years	2020	EDU	EDU unclear (CU) EDU low = high (EU)	No data	Unclear	Unclear	3	L

Authors	Data collection	Location	Participants	Study size	Measure(s) of SES	Main findings by SES measure(s) for SLT***	Main findings for combustible tobacco use (general SES)	Potential impact on smoking inequalities - Current Use	Potential impact on smoking inequalities - Ever Use	Evidence Tier	Critical Appraisal
Fischer et al. 2014	2012	Switzerland	mean age 19.5, men	5270	EDU, INC	EDU low > high<br>INC unclear	No data	Positive (EDU) Unclear (INC)	None	3	M
Grotvedt et al. 2008	2000-2004	Norway	14-18	15931	EDU, INC	INC unclear<br>EDU low > high	low > high	Positive (EDU) Unclear (INC)	None	1	M
Overland et al. 2010	2004, 2007	Norway	16-20	4815 (pooled)	EDU	EDU low = high	low > high	Neutral	None	1	M
Pedersen et al. 2014	2010	Norway	16-17	2796	EDU, INC	EDU unclear<br>INC unclear	Unclear	Unclear (EDU, INC)	None	3	L
Pedersen and Bakken 2016	2012	Norway, Oslo	15-17	6508	Composite index	high > low	Low > high	Negative	None	3	H
Wang et al. 1994*	1989	US	12-18 years	6599	INC	INC high > low	Low = high	Unclear	None	4	L

Authors	Data collection	Location	Participants	Study size	Measure(s) of SES	Main findings by SES measure(s) for SLT***	Main findings for combustible tobacco use (general SES)	Potential impact on smoking inequalities - Current Use	Potential impact on smoking inequalities - Ever Use	Evidence Tier	Critical Appraisal
Wang et al. 1998	1993	US	12-18 years	8725	EDU, INC	INC high > low (CU, EU)<br>EDU unclear (CU, EU)	Unclear	Unclear (EDU, INC)	Unclear (EDU, INC)	4	L

*Uses data from same survey as Wang et al 1998, but samples collected in different years.
***SLT, smokeless tobacco; EDU, education; INC, income; CU, current use; EU, ever use

Table 7: All nicotine replacement therapy studies

Authors	Data collection	Location	Study size	Measure(s) of SES	Main findings by SES measure(s) for NRT***	Potential impact on smoking inequalities - Current Use	Potential impact on smoking inequalities - Ever Use	Evidence Tier	Critical Appraisal
Alberg et al. 2005	1998	US, Washington County	1954	EDU	High EDU > low EDU	None	Positive	3	L
Clare et al. 2014	2001<br>2004<br>2007<br>2010	Australia	23402 (pooled)	EDU, INC	INC high > low<br>EDU unclear	Neutral (SEIFA)<br>Unclear (EDU)<br>Negative (INC)*	None	3	M

Kotz and West. 2009	2006-2008	UK, England	6950	Occupational group	low = high	Neutral*	None	3	M
Kushnir et al. 2017	Unclear	Canada	500	EDU, INC	EDU low > high<br>INC low > high	Neutral (EDU, INC)**	None	3	L

*Use for cessation in the last year.
**Use for cessation over a five week period.
***EDU, education; INC, income; CU, current use; EU, ever use
