# Supplementary material for: Potential for non-combustible nicotine products to reduce socioeconomic inequalities in smoking: a systematic review and synthesis of best available evidence
Source: BMC Public Health. 2019 Nov 6;19:1469. doi: 10.1186/s12889-019-7836-4 (PMC6836524; doi:10.1186/s12889-019-7836-4)
Supplement: Supplementary file 7 — Additional file 7. Full list of studies included in review https://documentcloud.adobe.com/link/track?uri=urn%3Aaaid%3Ascds%3AUS%3Ac6a4acca-8e43-4e7e-a5ad-cff8464de9b8 [file 12889_2019_7836_MOESM7_ESM.pdf]

Additional File 7: Full list of studies included in review

Paper: Potential for non-combustible nicotine products to reduce socioeconomic inequalities in smoking: a systematic review and synthesis of best available evidence.
Authors: Mark Lucherini, Sarah Hill, Katherine Smith

Accortt, N. A., Waterbor, J. W., Beall, C., Howard, G., & Brooks, M. (2005). Need to Educate Primary Caregivers About the Risk Factor Proile of Smokeless Tobacco Users. *Journal of Cancer Education*, *20*(4), 222–228.

Agaku, I., King, B., Husten, C., Bunnell, R., Ambrose, B., Hu, S., … Day, H. (2014). Tobacco Product Use Among Adults — United States, 2012–2013. *Morbidity & Mortality Weekly Report*, *63*(25), 542–547.

Alberg, A., Patnaik, J., May, J., Hoffman, S., Gitchell, J., Comstock, G., & Helzlsouer, K. (2005). Nicotine Replacement Therapy Use Among a Cohort of Smokers. *Journal of Addictive Diseases*, *21*(1), 101–113. http://doi.org/10.1300/J069v24n01

Babineau, K., Taylor, K., & Clancy, L. (2015). Electronic cigarette use among irish youth: A cross sectional study of prevalence and associated factors. *PLoS ONE*, *10*(5), 1–10. http://doi.org/10.1371/journal.pone.0126419

Backinger, C. L., Fagan, P., O'Connell, M. E., Grana, R., Lawrence, D., Bishop, J. A., & Gibson, J. T. (2008). Use of other tobacco products among U.S. adult cigarette smokers: Prevalence, trends and correlates. *Addictive Behaviors*, *33*(3), 472–489. http://doi.org/10.1016/j.addbeh.2007.10.009

Bhattacharyya, N. (2012). Trends in the use of smokeless tobacco in United States, 2000-2010. *Laryngoscope*, *122*(10), 2175–2178. http://doi.org/10.1002/lary.23448

Biener, L., McCausland, K., Curry, L., & Cullen, J. (2011). Prevalence of trial of snus products among adult smokers. *American Journal of Public Health*, *101*(10), 1874–1876. http://doi.org/10.2105/AJPH.2010.200097

Brown, J., West, R., Beard, E., Michie, S., Shahab, L., & McNeill, A. (2014). Prevalence and characteristics of e-cigarette users in Great Britain: Findings from a general population survey of smokers. *Addictive Behaviors*, *39*(6), 1120–1125. http://doi.org/10.1016/j.addbeh.2014.03.009

Carrieri, V., & Jones, A. M. (2016). Smoking for the poor and vaping for the rich? Distributional concerns for novel nicotine delivery systems. *Economics Letters*, *149*, 71–74. http://doi.org/10.1016/j.econlet.2016.10.012

Chang, J. T., Levy, D. T., & Meza, R. (2016). Trends and Factors Related to Smokeless Tobacco Use in the United States. *Nicotine & Tobacco Research*, *18*(8), 1740–1748. http://doi.org/10.1093/ntr/ntw090

Chivers, L. L., Hand, D. J., Priest, J. S., & Higgins, S. T. (2016). E-cigarette use among women of reproductive age: Impulsivity, cigarette smoking status, and other risk factors. *Preventive Medicine*, *92*, 126–134. http://doi.org/10.1016/j.ypmed.2016.07.029

Christensen, T., Welsh, E., & Faseru, B. (2014). Profile of e-cigarette use and its relationship with cigarette quit attempts and abstinence in Kansas adults. *Preventive Medicine*, *69*, 90–94. http://doi.org/10.1016/j.ypmed.2014.09.005

Clare, P., Slade, T., Courtney, R. J., Martire, K. a., & Mattick, R. P. (2014). Use of smoking cessation and quit support services by socioeconomic status over 10 years of the national drug strategy household survey. *Nicotine & Tobacco Researchesearch*, *16*(12), 1647–1655. http://doi.org/10.1093/ntr/ntu119

Douptcheva, N., Gmel, G., Studer, J., Deline, S., & Etter, J. F. (2013). Use of electronic cigarettes among young swiss men. *Journal of Epidemiology and Community Health*, *67*(12), 1075–1076. http://doi.org/10.1136/jech-2013-203152

Engström, K., Magnusson, C., & Galanti, M. R. (2010). Socio-demographic, lifestyle and health characteristics among snus users and dual tobacco users in Stockholm County, Sweden. *BMC Public Health*, *10*(1), 619. http://doi.org/10.1186/1471-2458-10-619

Fischer, R., Clair, C., Studer, J., Cornuz, J., & Gmel, G. (2014). Prevalence and factors associated with use of smokeless tobacco in young Swiss men. *European Journal of Public Health*, *24*(3), 459–464. http://doi.org/10.1093/eurpub/ckt086

Gallus, S., Lugo, A., Pacifici, R., Pichini, S., Colombo, P., Garattini, S., & La Vecchia, C. (2014). E-cigarette awareness, use, and harm perceptions in Italy: a national representative survey. *Nicotine & Tobacco Research*, *16*(12), 1541–1548. http://doi.org/10.1093/ntr/ntu124

Grotvedt, L., Stigum, H., Hovengen, R., & Graff-Iversen, S. (2008). Social differences in smoking and snuff use among Norwegian adolescents: A population based survey. *BMC Public Health*, *8*, 1–12. http://doi.org/10.1186/1471-2458-8-322

Hill, M. E., Harrell, J. S., & McCormick, L. K. (1992). Predictors of smokeless tobacco use by adolescents. *Research in Nursing & Health*, *15*(5), 359.

Hu, S. S., Neff, L., Agaku, I. T., Cox, S., Day, H. R., Holder-Hayes, E., & King, B. a. (2016). Tobacco Product Use Among Adults — United States, 2013–2014. *MMWR. Morbidity and Mortality Weekly Report*, *65*(27), 685–691. http://doi.org/10.15585/mmwr.mm6527a1

Huang, J., Kim, Y., Vera, L., & Emery, S. L. (2016). Electronic Cigarettes among Priority Populations: Role of Smoking Cessation and Tobacco Control Policies. *American Journal of Preventive Medicine*, *50*(2), 199–209. http://doi.org/10.1016/j.amepre.2015.06.032

Khoury, M., Manlhiot, C., Fan, C. P. S., Gibson, D., Stearne, K., Chahal, N., … McCrindle, B. W. (2016). Reported electronic cigarette Use among adolescents in the Niagara region of Ontario. *CMAJ : Canadian Medical Association Journal*, *188*(11), 794–800. http://doi.org/10.1503/cmaj/151169

King, B. a., Alam, S., Promoff, G., Arrazola, R., & Dube, S. R. (2013). Awareness and ever-use of electronic cigarettes among U.S. adults, 2010-2011. *Nicotine and Tobacco Research*, *15*(9), 1623–1627. http://doi.org/10.1093/ntr/ntt013

King, B. a., Patel, R., Nguyen, K. H., & Dube, S. R. (2015). Trends in awareness and use of electronic cigarettes among US adults, 2010-2013. *Nicotine and Tobacco Research*, *17*(2), 219–227. http://doi.org/10.1093/ntr/ntu191

Kinnunen, J. M., Ollila, H., Lindfors, P. L., & Rimpelä, A. H. (2016). Changes in electronic cigarette use from 2013 to 2015 and reasons for use among finnish adolescents. *International Journal of Environmental Research and Public Health*, *13*(11). http://doi.org/10.3390/ijerph13111114

Kotz, D., & West, R. (2009). Explaining the social gradient in smoking cessation: It's not in the trying, but in the succeeding. *Tobacco Control*, *18*(1), 43–46. http://doi.org/10.1136/tc.2008.025981

Kushnir, V., Sproule, B. a., & Cunningham, J. a. (2017). Mailed distribution of free nicotine patches without behavioral support: Predictors of use and cessation. *Addictive Behaviors*, *67*, 73–78. http://doi.org/10.1016/j.addbeh.2016.12.008

Kvaavik, E., Lund, I., Nygård, M., & Hansen, B. T. (2016). *Lifestyle Correlates of female snus use and smoking: A large population-based survey of women in Norway*. *Nicotine and Tobacco Research* (Vol. 18). http://doi.org/10.1093/ntr/ntv126

Mazurek, J. M., Syamlal, G., King, B. a, Castellan, R. M., Division of Respiratory Disease Studies, N. I. for O. S., & Health, C. D. C. (2014). Smokeless tobacco use among working adults - United States, 2005 and 2010. *MMWR - Morbidity & Mortality Weekly Report*, *63*(22), 477–482.

McMillen, R., Maduka, J., & Winickoff, J. (2012). Use of emerging tobacco products in the United States. *Journal of Environmental and Public Health*. http://doi.org/10.1155/2012/989474

Mushtaq, N., Williams, M. B., & Beebe, L. a. (2012). Concurrent use of cigarettes and smokeless tobacco among US males and females. *Journal of Environmental and Public Health*. http://doi.org/10.1155/2012/984561

Nelson, D. E., Tomar, S. L., Mowery, P., & Siegel, P. Z. (1996). Trends in smokeless tobacco use among men in four states, 1988 through 1993. *American Journal of Public Health*, *86*(9), 1300–1303. http://doi.org/10.2105/AJPH.86.9.1300

Norberg, M., Malmberg, G., Ng, N., & Broström, G. (2011). Who is using snus? - Time trends, socioeconomic and geographic characteristics of snus users in the ageing Swedish population. *BMC Public Health*, *11*. http://doi.org/10.1186/1471-2458-11-929

Novotny, T., Pierce, J., Fiore, M., & Davis, R. (1989). Smokeless tobacco use in the United States: the adult use of tobacco surveys. *NCI Monographs: A Publication of the National Cancer Institute*, *8*, 25–28.

Øverland, S., Tjora, T., Hetland, J., & Aarø, L. E. (2010). Associations between adolescent socioeducational status and use of snus and smoking. *Tobacco Control*, *19*(4), 291–296. http://doi.org/10.1136/tc.2009.034512

Pearson, J. L., Richardson, A., Niaura, R. S., Vallone, D. M., & Abrams, D. B. (2012). E-cigarette awareness, use, and harm perceptions in US adults. *American Journal of Public Health*, *102*(9), 1758–1766. http://doi.org/10.2105/AJPH.2011.300526

Pedersen, W., & Bakken, A. (2016). Urban landscapes of adolescent substance use. *Acta Sociologica*, *59*(2), 131–150. http://doi.org/10.1177/0001699315625448

Pedersen, W., & von Soest, T. (2014). Tobacco use among Norwegian adolescents: From cigarettes to snus. *Addiction*, *109*(7), 1154–1162. http://doi.org/10.1111/add.12509

Regan, A. K., Promoff, G., Dube, S. R., & Arrazola, R. (2013). Electronic nicotine delivery systems: Adult use and awareness of the “e-cigarette” in the USA. *Tobacco Control*, *22*(1), 19–23. http://doi.org/10.1136/tobaccocontrol-2011-050044

Rennie, L. J., Bazillier-Bruneau, C., & Rouëssé, J. (2016). Harm Reduction or Harm Introduction? Prevalence and Correlates of E-Cigarette Use among French Adolescents. *Journal of Adolescent Health*, *58*(4), 440–445. http://doi.org/10.1016/j.jadohealth.2015.12.013

Roberts, M. E., Doogan, N. J., Kurti, A. N., Redner, R., Gaalema, D. E., Stanton, C. a., … Higgins, S. T. (2016). Rural tobacco use across the United States: How rural and urban areas differ, broken down by census regions and divisions. *Health and Place*, *39*, 153–159. http://doi.org/10.1016/j.healthplace.2016.04.001

Rodu, B., & Cole, P. (2009). Smokeless tobacco use among men in the United States, 2000 and 2005. *Journal of Oral Pathology & Medicine*, *38*(7), 545–550. http://doi.org/10.1111/j.1600-0714.2009.00780.x

Romito, L., & Saxton, M. K. (2014). Impact of promotions on awareness, trial, and likelihood of trial of new dissolvable tobacco. *American Journal of Health Promotion*, *28*(4), 251–258. http://doi.org/10.4278/ajhp.120926-QUAN-469

Sung, H. Y., Wang, Y., Yao, T., Lightwood, J., & Max, W. (2016). Polytobacco use of cigarettes, cigars, chewing tobacco, and snuff among US adults. *Nicotine and Tobacco Research*, *18*(5), 817–826. http://doi.org/10.1093/ntr/ntv147

Syamlal, G., Jamal, A., King, B. a., & Mazurek, J. M. (2016). Electronic Cigarette Use Among Working Adults — United States, 2014. *MMWR. Morbidity and Mortality Weekly Report*, *65*(22), 557–561. http://doi.org/10.15585/mmwr.mm6522a1

Syamlal, G., Jamal, A., & Mazurek, J. M. (2016). Combustible Tobacco and Smokeless Tobacco Use Among Working Adults—United States, 2012 to 2014. *Journal of Occupational and Environmental Medicine*, *58*(12), 1185–1189. http://doi.org/10.1097/JOM.0000000000000898

Thorne, S., McClave, A., Rock, V., & Asman, K. (2010). Any Tobacco Use in 13 States — Behavioral Risk Factor Surveillance System, 2008. *MMWR. Morbidity and Mortality Weekly Report*, *59*(30), 946–950.

Vijayaraghavan, M., Pierce, J. P., White, M., & Messer, K. (2014). Differential use of other tobacco products among current and former cigarette smokers by income level. *Addictive Behaviors*, *39*, 1452–1458. http://doi.org/10.1016/j.addbeh.2014.05.029

Wang, M. Q., Fitzhugh, E. C., Eddy, J. M., Westerfield, R. C., & Fu, Q. (1998). Tobacco Use among School Adolescents: National Sociodemographic Risk Profiles. *Journal of Health Education*, *29*(3), 174–178. http://doi.org/10.1080/10556699.1998.10603330

Wang, M. Q., Fitzhugh, E. C., Green, L., Eddy, J. M., & Westerfield, R. C. (1994). Tobacco Use Among American Adolescents: Geographic and Deomographic Variations. *Southern Medical Journal*, *87*(6), 607–610.

Weaver, S. R., Majeed, B. A., Pechacek, T. F., Nyman, A. L., Gregory, K. R., & Eriksen, M. P. (2016). Use of electronic nicotine delivery systems and other tobacco products among USA adults, 2014: results from a national survey. *International Journal of Public Health*, *61*(2), 177–188. http://doi.org/10.1007/s00038-015-0761-0

White, T. J., Redner, R., Bunn, J. Y., & Higgins, S. T. (2016). Do Socioeconomic Risk Factors for Cigarette Smoking Extend to Smokeless Tobacco Use? *Nicotine and Tobacco Research*, *18*(5), 869–873. http://doi.org/10.1093/ntr/ntv199

Wills, T. a., Knight, R., Sargent, J. D., Gibbons, F. X., Pagano, I., & Williams, R. J. (2017). Longitudinal study of e-cigarette use and onset of cigarette smoking among high school students in Hawaii. *Tobacco Control*, *26*(1), 34–39. http://doi.org/10.1136/tobaccocontrol-2015-052705

Wilson, F. a., & Wang, Y. (2017). Recent Findings on the Prevalence of E-Cigarette Use Among Adults in the U.S. *American Journal of Preventive Medicine*, *52*(3), 385–390. http://doi.org/10.1016/j.amepre.2016.10.029
